# Behçet’s disease risk association fine-mapped on the *IL23R*–*IL12RB2* intergenic region in Koreans

**DOI:** 10.1186/s13075-017-1435-5

**Published:** 2017-10-10

**Authors:** Eun Ha Kang, Sewon Kim, Min Young Park, Ji Yong Choi, In Ah Choi, Min Jung Kim, You-Jung Ha, Eun Young Lee, Yun Jong Lee, Eun Bong Lee, Changwon Kang, Yeong Wook Song

**Affiliations:** 1 Division of Rheumatology, Department of Internal Medicine, Seoul National University Bundang Hospital, Seongnam, South Korea; 2 Department of Biological Sciences, Korea Advanced Institute of Science and Technology, Daejeon, South Korea; 3 DNA Link, Inc, Seoul, South Korea; 4 Division of Rheumatology, Department of Internal Medicine, Seoul National University Hospital, Seoul, South Korea; 5 Division of Rheumatology, Department of Internal Medicine, Chungbuk National University Hospital, Daejeon, South Korea; 6 Department of Molecular Medicine and Biopharmaceutical Sciences, Graduate School of Convergence Science and Technology, and College of Medicine, Seoul National University, Seoul, South Korea

**Keywords:** Behçet’s disease, Case-control disease-association study, *ERAP1*, Genotyping, Imputation, *IL10*, *IL12RB2*, *IL23R*, *STAT4*

## Abstract

**Background:**

Behçet’s disease (BD) susceptibility had been associated with single-nucleotide polymorphisms (SNPs) in *IL23R*–*IL12RB2*, *IL10, STAT4,* or *ERAP1* locus in Japanese, Turkish, Chinese, and other populations, but not in a Korean genome-wide association study (GWAS). We aimed to fine-map BD risk association of these four loci using extensive imputation and additional genotyping for replication.

**Methods:**

In the discovery phase, 369 patients with BD enrolled in the previous Korean GWAS and 2000 controls retrieved from a population-based cohort of healthy Koreans were imputed for their genotypes of all SNPs in the four loci using the Asian data of the 1000 Genomes Project as reference. For genotype imputation of *ERAP1* SNPs, the adjacent *ERAP2* SNPs were also covered. For the 10 most significantly associated SNPs (8 imputed and 2 GWAS-genotyped), an additional 84 patients with BD and 283 healthy controls were genotyped for replication. The results from the discovery and replication phases were pooled for meta-analysis using the Mantel-Haenszel test to estimate the odds ratio (OR) and 95% confidence interval (CI).

**Results:**

An *IL23R*–*IL12RB2* intergenic SNP rs1495965 was significantly associated with BD risk (OR (95% CI) = 1.5 (1.3, 1.7), *P* = 2.5 × 10^−7^) in the pooled meta-analysis of the discovery (1.4 (1.2, 1.7), *P* = 4.9 × 10^−7^) and replication (1.9 (1.3, 2.6), *P* = 6.0 × 10^−4^) phases. BD risk association was fine-mapped on the intergenic region rather than the two flanking genes, as rs1495966 and rs4655535, almost perfectly correlated with rs1495965 (*r* ^2^ = 0.99), were also located in the same intergenic region. Consistent with previous reports, the *P* values tended to be lower within *IL23R* than *IL12RB2*. On the other hand, several *IL10* SNPs were suggested for association in the discovery phase but all failed in the replication phase. No SNP in *ERAP1*–*ERAP2* and *STAT4* was suggested even in the discovery phase.

**Conclusions:**

BD susceptibility association was fine-mapped on the intergenic region between *IL23R* and *IL12RB2* as marked by three correlated SNPs, rs1495965, rs1495966, and rs4655535.

**Electronic supplementary material:**

The online version of this article (doi:10.1186/s13075-017-1435-5) contains supplementary material, which is available to authorized users.

## Background

Behçet’s disease (BD) is a chronic relapsing inflammatory disease characterized by orogenital ulcers, cutaneous inflammation and uveitis. In addition to its typical muco-cutaneous and ocular manifestations, BD is a multi-system disease that also targets musculoskeletal, vascular, nervous, and gastrointestinal systems [1]. Although the etiology of BD remains unclear, it is well-established that BD is strongly associated with HLA-B*51.

Since two landmark genome-wide association studies (GWAS) performed on Japanese and Turkish populations [2, 3] identified *HLA-A, IL10*, and *IL23R–IL12RB2* to be novel BD susceptibility loci, the association of *IL10* and *IL23R–IL12RB2* with BD has been replicated thereafter in various ethnic groups including the Chinese Han, Iranian, and Western Algerian populations [4–9]. The identification of *IL10* and *IL23R–IL12RB2* not only indicates involvement of non-HLA genes but also implies the importance of cytokine dysregulation in the pathogenesis of BD.

Additional BD susceptibility loci other than *IL10* and *IL23R–IL12RB2* identified by subsequent GWAS include *STAT4* in the Chinese Han [10] and *GIMAP* in Koreans [11]. STAT4 is a transcription factor that transduces IL-12, IL-23, and type 1 interferon signals in T cells and monocytes [12]. Thus, the functional relevance of *STAT4* in BD appears sensible because T helper (Th) 1 and Th17 cytokines are closely related to BD pathogenesis [13–15]. Notably, the Korean GWAS, which identified association between BD and *GIMAP*, failed to replicate the association with *IL10*, *IL23R*–*IL12RB2,* or *STAT4* [11]. This lack of association could have been due to insufficient statistical power of the study (limitation in sample size or SNP density) or due to the unique ethnic background.

The imputation technique, coupled with the GWAS database, has been successfully used in studying BD genetics to achieve genome-wide fine-mapping. By enhancing SNP density and thereby helping identify the most strongly associated among virtually all SNPs in a region of interest, genotype imputation can upgrade the statistical power of GWAS. Applying this technique in the GWAS database has enabled identification of the association with *STAT4* in the Turkish and Japanese populations and recessive association with two nonsynonymous *ERAP1* SNPs in the Turkish population [16]. Their minor alleles were too few in the Japanese population to evaluate the recessive effect. *ERAP1* homozygotes of the BD risk-associated allele conferred the risk preferentially to HLA-B*51 positive individuals, suggesting a gene-gene interaction between *ERAP1* and HLA-B*51.

We hypothesized that those susceptibility genes identified in other Asian groups are associated with BD in Koreans as well and selected the genetic regions to be imputed, where their association has been confirmed in at least two Asian groups at GWAS level sample size. *ERAP1* was also selected to examine a gene-gene interaction with HLA-B*51 in our population. Finally, we aimed to fine-map *IL23R*–*IL12RB2*, *IL10,* and *STAT4* regions and *ERAP1* by applying imputation technique to our Korean GWAS dataset.

## Methods

### Study participants

A total of 369 Korean patients with BD (cases) enrolled in a previous GWAS [11] and 2000 age-matched and sex-matched controls retrieved from a population-based cohort of healthy Koreans (Korea Biobank Network, http://cdc.co.kr) were included in this study. For replication, a different set of 84 Korean patients with BD and 283 age-matched and sex-matched healthy controls who had not been included in the discovery phase were recruited. Patients with BD fulfilled the International Study Group diagnostic criteria for BD [17].

### Genotype imputation in the discovery phase

Using the Asian dataset (CHB + JPT) of the 1000 Genomes Project as a reference panel, the missing genotypes in the four loci were inferred for 369 cases and 2000 controls after phasing the observed genotypes derived from the Korean GWAS data [11]. Before phasing, the GWAS genotypes determined by the Affymetrix genome-wide human SNP array 6.0 were screened for quality control in terms of call rate (>95%), minor allele frequency (>5%), and Hardy-Weinberg equilibrium *P* value in controls (>0.0001). Boundaries of imputation ranges were determined to include linkage disequilibrium (LD) blocks where the qualified GWAS SNPs of a given gene reside.

For *IL10*, SNPs with minor allele frequency of 1–5% were additionally included due to the paucity of GWAS SNPs in this region if they satisfy the other quality control measures and if their signal clusters showed correct call decisions in manual inspection. *ERAP2* SNPs were included for high-quality imputation of *ERAP1* SNP genotypes since the LD block expanded from *ERAP1* to *ERAP2*. The MATCH v1.0.16 software (University of Michigan, Ann Arbor, MI, USA) was used to perform the imputation. While 109 SNPs of the four loci had been genotyped in the previous GWAS, 1629 additional SNPs were imputed and passed a cutoff of imputation quality, *r* square (Rsq) > 0.3 in this study.

### Direct genotyping in the replication phase

Among the imputed SNPs, two *IL10* SNPs with a lower *P* value for BD risk association than the GWAS SNP rs1554286 and six *IL23R–IL12RB2* SNPs with a lower or similar *P* value than the GWAS SNP rs6677188 were selected together with the two GWAS SNPs for a replication study. Thus, 10 lead SNPs (3 in *IL10* and 7 in *IL23R–IL12RB2*) were genotyped in an additional population of 84 BD cases and 283 controls using the Taqman® primers and probes designed by Applied Biosystems (Foster City, CA, USA).

### Statistical analysis for BD association

The chi-square test or Fisher’s exact test was used to compare the allele frequencies between patient cases and healthy controls. Statistical analysis was done using the SPSS v17.0 software (SPSS Inc., Chicago, IL, USA). For meta-analysis, data were pooled and analyzed according to the Mantel-Haenszel test using the Stata v14 software (StataCrop LP, College Station, TX, USA). Between-study heterogeneity was quantified using the *I* ^2^ statistic.

## Results

### Clinical characteristics of study participants

The clinical manifestations of patients with BD in the discovery and replication phases are separately summarized in Table 1. Compared to the GWAS population enrolled in the discovery phase, the replication phase population had more women (57% vs. 50%), more skin lesions (96.3% vs. 89.2% for any skin lesion, *P* = 0.04; and 61.4% vs. 54.6% positivity in pathergy testing), but fewer eye lesions (28.8% vs 43.5%, *P* = 0.01). Central nervous system involvement was more frequent in the discovery phase (11.6% vs. 3.8%, *P* = 0.03).

**Table 1:** Clinical characteristics of the enrolled patients with Behçet’s disease

Characteristic	Discovery phase (n = 379)	Replication phase (n = 84)	*P* value^b^
Male (%): female	191 (50): 188	36 (43): 48	
Age at diagnosis of BD (years)^a^	41.6 ± 10.1	44.1 ± 11.4	
Clinical manifestation (%)			
Recurrent oral ulcer	100	100	
Recurrent genital ulcer	74.4	80.0	
Skin lesions	89.2	96.3	0.04
Eye lesions	43.5	28.8	0.01
Positive pathergy test	54.6 (130/238)	61.4 (27/44)	
Vascular involvement	16.4	17.5	
Central nervous system involvement	11.6	3.8	0.03
Joint involvement	41.4	51.3	

*BD* Behçet’s disease

^a^Age is presented as mean ± standard deviation

^b^Only *P* values <0.05 are presented

### LD structure comparison in Asian populations

In order to see whether the 1000 Genomes Project data can be used as reference for imputing the Korean genotypes of the four loci, we first examined similarity of LD structures of HapMap-scale SNPs in the loci between the Korean genotype data of the Korean HapMap Project (equivalent to the International HapMap Project) and the Chinese and Japanese (referred to as Asian) sequence data of the 1000 Genomes Project.

The correlation coefficient (*r* ^2^) was calculated for all SNPs within each of the four loci in reference to the SNP that was most significant in the Korean GWAS (rs6677188 in *IL23R*–*IL12RB2*, rs1554286 in *IL10*, rs1031508 in *STAT4*, and rs26652 in *ERAP1*–*ERAP2*) using the Korean genotype data and Asian sequence data separately. Then, SNP distributions plotted in descending order of *r* ^2^ values were compared between the two populations (Additional file 1: Figure S1). The two plots were nearly superimposable across all SNPs in each of *IL10, IL12RB–IL23R,* and *STAT4* loci and across the highly correlated SNPs (0.5 ≤ *r* ^2^ ≤ 1.0) in *ERAP1–ERAP2*.

### Association analysis in the discovery phase

**Fig. 1:**
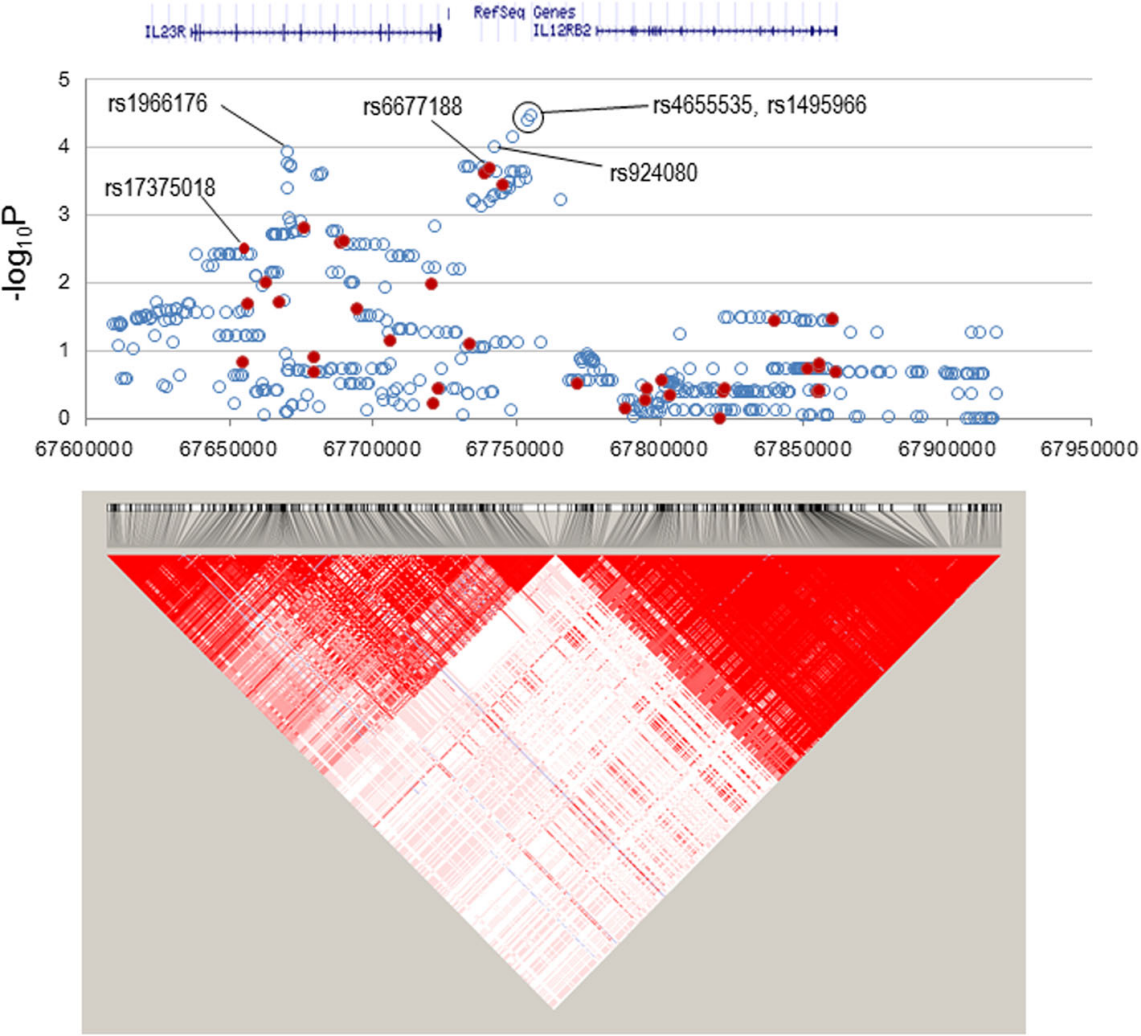
Manhattan plot for the *IL23R–IL12RB2* locus. All imputed (solid red circles) and genome-wide association study (GWAS)-genotyped (open blue circles) SNPs are plotted together with a linkage disequilibrium structure below

In the *IL23R–IL12RB2* locus ranging from − 22.7 kb of *IL23R* to + 54.3 kb of *IL12RB2*, 37 SNPs had been genotyped in the previous GWAS [11] and 509 additional SNPs were imputed in this study for association tests (Fig. 1). The most significantly associated SNP was rs4655535 (imputed, OR = 1.4 (1.2, 1.7), *P* = 0.000033), which had a sixfold lower *P* value than the GWAS-genotyped SNP rs6677188 (*P* = 0.00020), which had the same OR and 95% CI (Table 2).

**Table 2:** Allelic association of SNPs after imputation in the discovery phase

Locus	SNP	Risk allele (%)		HWE *P*	Rsq^c^	Allelic association	
		BD cases (n = 738)	Controls (n = 4000)			OR (95% CI)	*P*
*IL23R*–*IL12RB2*	rs4655535 (*G* > *T*)^a^	467 (63)	2201 (55)	0.00023	0.40	1.4 (1.2, 1.7)	0.000033
	rs1495966 (*T* > *C*)^a^	467 (63)	2204 (55)	0.00042	0.40	1.4 (1.2, 1.7)	0.000038
	rs1495965 (*C* > *T*)^a^	466 (63)	2203 (55)	0.00050	0.40	1.4 (1.2, 1.7)	0.000049
	rs6665569 (*T* > *C*)^a^	465 (53)	2210 (55)	0.047	0.48	1.4 (1.2, 1.6)	0.000094
	rs1966176 (*G* > *A*)^a^	506 (69)	2442 (61)	0.61	0.87	1.4 (1.2, 1.7)	0.00011
	rs6677188 (*T* > *A*)^a,b^	588 (80)	2919 (73)	0.034	NA	1.4 (1.2, 1.8)	0.00020
	rs924080 (*T* > *C*)^a^	590 (80)	2940 (73)	0.11	0.77	1.4 (1.2, 1.7)	0.00022
*IL10*	rs1518110 (*A* > *C*)	563 (76)	2728 (68)	0.47	0.91	1.5 (1.2, 1.8)	0.000012
	rs1518111 (*T* > *C*)^a^	563 (76)	2728 (68)	0.47	0.91	1.5 (1.2, 1.8)	0.000012
	rs1800871 (*A* > *G*)	563 (76)	2728 (68)	0.47	0.87	1.5 (1.2, 1.8)	0.000012
	rs1800872 (*T* > *G*)^a^	563 (76)	2728 (68)	0.47	0.87	1.5 (1.2, 1.8)	0.000012
	rs3024490 (*A* > *C*)	563 (76)	2728 (68)	0.47	0.85	1.5 (1.2, 1.8)	0.000012
	rs1554286 (*A* > *G*)^a,b^	551 (76)	2720 (68)	0.47	NA	1.5 (1.2, 1.8)	0.000030

*SNP* single-nucleotide polymorphism, *BD* Behçet’s disease, *HWE* Hardy-Weinberg equilibrium, *Rsq r* square, *OR* odds ratio, *CI* confidence interval, *NA* not applicable

^a^The ten SNPs were genotyped in the subsequent replication phase

^b^The two SNPs had been genotyped in the previous genome-wide association study [11] and the genotypes of the others were imputed in this study

^c^Rsq is an imputation quality metric estimated for each imputed SNP

Furthermore, four other SNPs (rs1495966, rs1495965, rs6665569, and rs1966176 in ascending order of *P* value) had lower *P* values (0.000038 ≤ *P* ≤ 0.00011) and rs924080 had a similar *P* value (*P* = 0.00022) compared to rs6677188. These SNPs were all located within the intergenic region except for rs1966176 located within the *IL23R* gene as shown in Fig. 1. Subsequently, seven SNPs (rs4655535, rs1495966, rs1495965, rs6665569, rs1966176, rs924080, and rs6677188) were chosen to be genotyped for replication of BD risk association. As seen in Turkish and Iranian studies [3, 8], many SNPs located within the *IL23R* gene had lower *P* values than any SNP located within the *IL12RB2* gene, although it needs to be verified whether the BD risk-associated intergenic SNPs affect expression of *IL23R*, *IL12RB2*, or both in future studies.

**Fig. 2:**
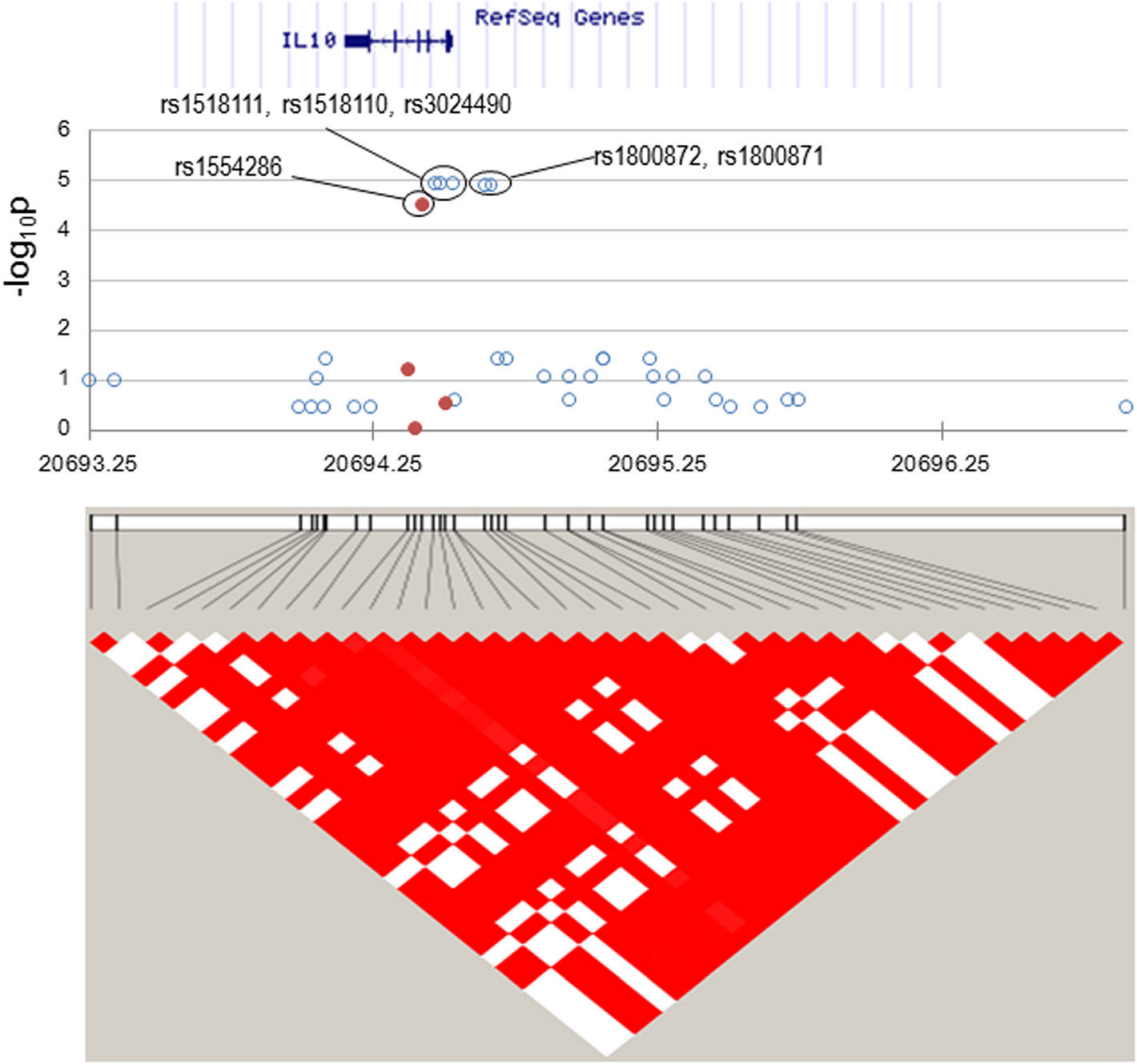
Manhattan plot for the *IL10* locus. All imputed (solid red circles) and genome-wide association study (GWAS)-genotyped (open blue circles) SNPs are plotted together with a linkage disequilibrium structure below

In the *IL10* locus ranging from − 8.4 kb to + 23.1 kb, four SNPs had been genotyped [11] and 34 SNPs were imputed (Fig. 2). The most significantly associated SNPs were rs1518110, rs1518111, rs1800871, rs1800872, and rs3024490 (all imputed, OR (95% CI) = 1.5 (1.2, 1.8), *P* = 0.000012), which were perfectly correlated (*r* ^2^ = 1.0) with each other (Table 2). These five SNPs had only a slightly lower *P* value (0.000030) and the same OR (95% CI) (1.5 (1.2, 1.8)]), compared to the GWAS-genotyped rs1554286 (Table 2). Among these six highly correlated SNPs, genotyping of any one would be sufficient for the replication study, but three SNPs, rs1518111, rs1800872, and rs1554286, were genotyped in the replication phase just for redundant assurance.

**Fig. 3:**
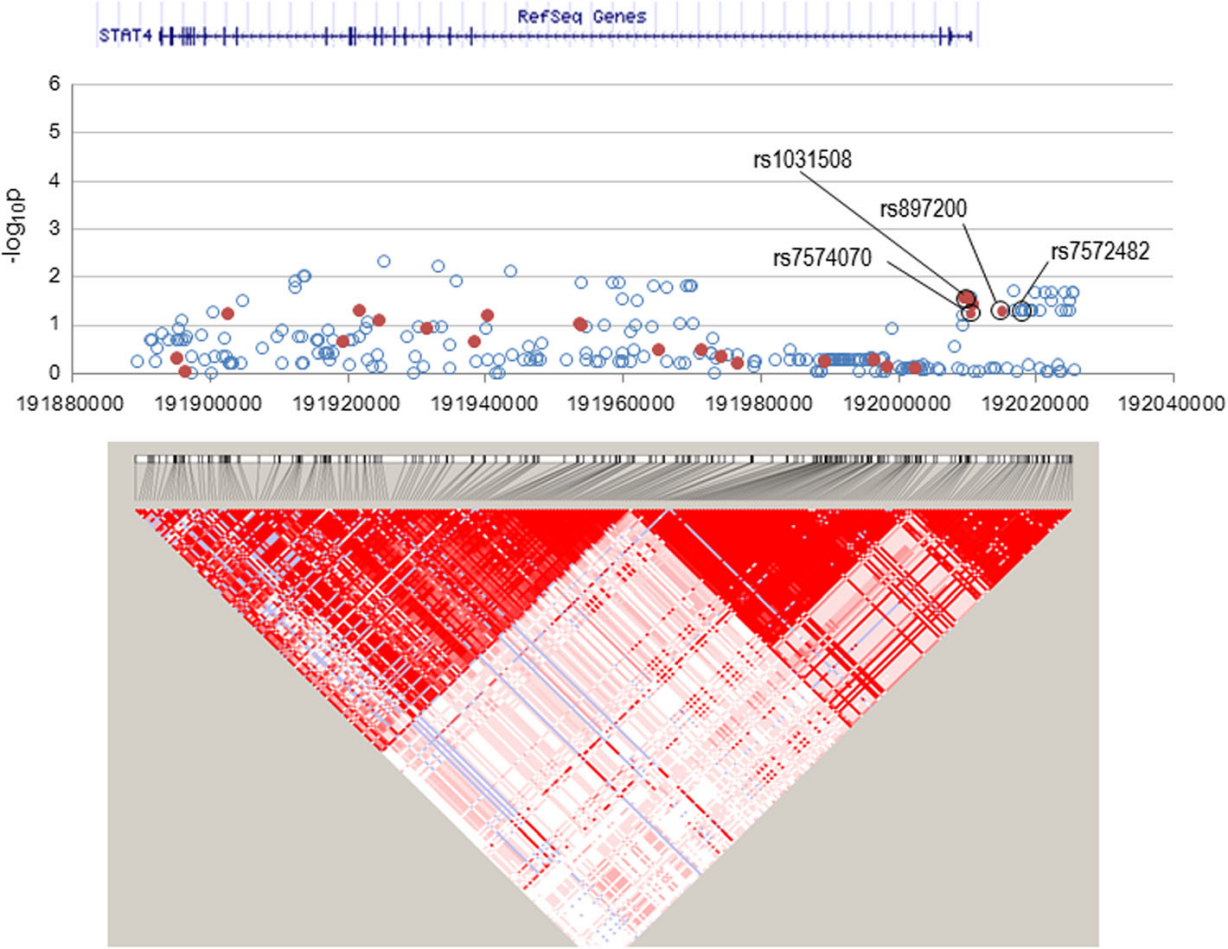
Manhattan plot for *STAT4* locus. All imputed (solid red circles) and genome-wide association study (GWAS)-genotyped (open blue circles) SNPs are plotted together with a linkage disequilibrium structure below

**Fig. 4:**
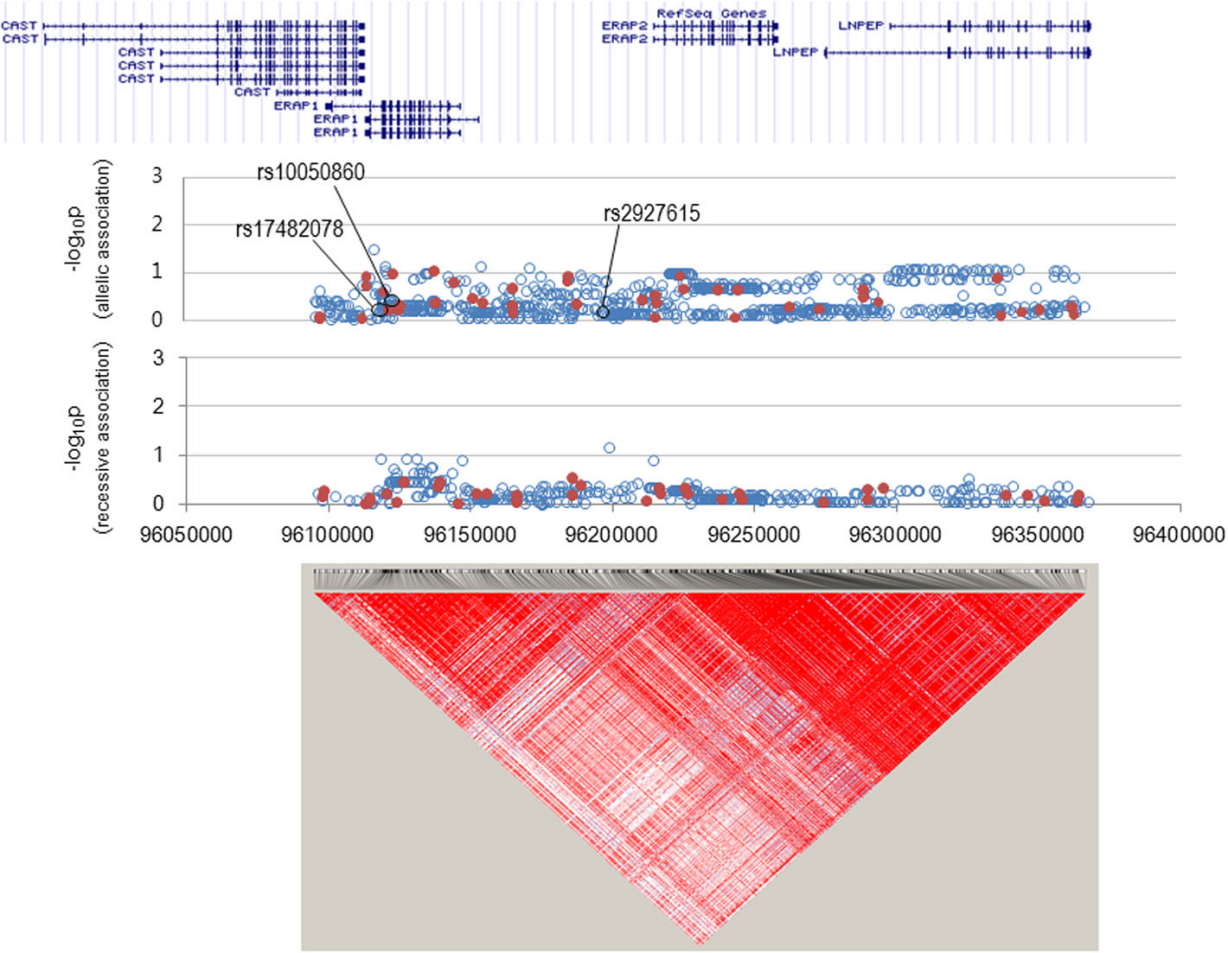
Manhattan plot for the *ERAP1–ERAP2* locus. All imputed (solid red circles) and genome-wide association study (GWAS)-genotyped (open blue circles) SNPs are plotted together with a linkage disequilibrium structure below. The top panel represents the allelic association and the bottom one represents the recessive association

In the *STAT4* locus ranging from − 4.7 kb to + 9.4 kb, 25 SNPs had been genotyped [11] and 239 SNPs were imputed, but no SNPs were associated with BD risk in this Korean population (Fig. 3). In the *ERAP1–ERAP2* locus ranging from − 14.0 kb of *ERAP1* to + 112.0 kb of *ERAP2*, 43 SNPs had been genotyped [11] and 847 SNPs were imputed but no SNPs were associated (Fig. 4). Minor allele homozygotes of two *ERAP1* SNPs, rs17482078, and rs10050860, had been associated with Turkish BD [16], but their minor allele frequencies were similar between BD cases and controls (4.9% vs. 4.4% for rs17482078; 4.9% vs. 4.5% for rs10050860, respectively) without such homozygote carriers in this study.

### Association analysis in the replication phase

The above-mentioned seven *IL23R*–*IL12RB2* SNPs and three *IL10* SNPs were genotyped in a replication population consisting of 89 BD cases and 283 healthy controls of Korean ethnicity (Table 3). All SNPs in *IL23R*–*IL12RB2* maintained BD risk association with *P* values (0.00062 ≤ *P* ≤0.0049) lower than the significance level for multiple testing, α = 0.05/10 = 0.005 (for 10 SNPs), except for rs6665569 (*P* = 0.0063). The OR values of these seven SNPs were higher with experimentally determined genotype data in the replication phase (1.6 ≤ OR ≤2.1) than with imputed genotype data in the discovery phase (OR = 1.4), although their 95% CI overlapped.

**Table 3:** Allelic association of SNPs in the replication phase

Locus	SNP	BD cases, risk allele/total (%)	Controls, risk allele/total (%)	Allelic association	
				OR (95% CI)	*P*
*IL23R*–*IL12RB2*	rs1495965 (*C* > *T*)	106/168 (63)	272/566 (48)	1.9 (1.3, 2.6)	0.00062
	rs1495966 (*T* > *C*)	106/168 (63)	264/548 (48)	1.8 (1.3, 2.6)	0.00071
	rs4655535 (*G* > *T*)	107/168 (64)	277/566 (49)	1.8 (1.3, 2.6)	0.00078
	rs924080 (*T* > *C*)	143/168 (85)	413/566 (73)	2.1 (1.3, 3.4)	0.0013
	rs1966176 (*G* > *A*)	118/168 (70)	322/562 (57)	1.8 (1.2, 2.6)	0.0026
	rs6677188 (*T* > *A*)	142/168 (85)	419/566 (74)	1.9 (1.2, 3.0)	0.0049
	rs6665569 (*T* > *C*)	103/168 (61)	271/550 (49)	1.6 (1.2, 2.3)	0.0063
*IL10*	rs1518111 (*A* > *G*)	120/168 (71)	408/566 (72)	1.0 (0.7, 1.4)	0.87
	rs1800872 (*A* > *C*)	120/168 (71)	408/566 (72)	1.0 (0.7, 1.4)	0.87
	rs1554286 (*T* > *C*)	120/168 (71)	396/554 (71)	1.0 (0.7, 1.5)	0.99

*SNP* single-nucleotide polymorphism, *BD* Behçet’s disease, *OR* odds ratio, *CI* confidence interval

However, the association between the three *IL10* SNPs and risk of BD was no longer significant in the replication phase, as the *P* values were much higher than a marginal significance level of α = 0.05 (Table 3). Association test results were the same for the three highly correlated SNPs when they were re-genotyped for 20 randomly chosen cases of BD and 42 controls, with alternatively designed primers and probes in a blinded manner, rejecting any possibility of technical or sampling errors in genotyping.

### Meta-analysis

The discovery and replication phase data of the seven lead SNPs in the *IL23R–IL12RB2* locus were combined for meta-analysis (Table 4). All seven SNPs were significantly associated with BD susceptibility, as their *P* values passed a significance level for multiple testing of all 1629 imputed and 109 genotyped SNPs, α = 0.05/1738 = 2.9 × 10^−5^: the *P* value was an order of magnitude lower for three SNPs, rs1495965 (*P* = 2.5 × 10^−7^), rs4655535 (*P* = 2.6 × 10^−7^), and rs1495966 (*P* = 2.9 × 10^−7^), than the other four SNPs (*P* ≥ 2.1 × 10^−6^). The three lowest *P* value SNPs were almost perfectly correlated with each other (*r* ^2^ = 0.99) and all located in the intergenic region between *IL23R* and *IL12RB2*.

**Table 4:** Meta-analysis on allelic associations of *IL23R*–*IL12RB2* SNPs

SNP	Phase	BD cases, risk allele/total	Controls, risk allele/total	Weight %	*I* ^2^ %	OR (95% CI)	*P*
rs1495965	Dis.	467/738 (63)	2203/4000 (55)	83.7	35	1.41 (1.20, 1.65)	0.000049
	Rep.	106/168 (63)	272/566 (48)	16.3		1.85 (1.30, 2.63)	0.00060
	Total	573/906 (63)	2475/4566 (54)			1.47 (1.27, 1.70)	0.00000025
rs4655535	Dis.	467/738 (63)	2201/4000 (55)	83.7	27	1.41 (1.20, 1.66)	0.000033
	Rep.	107/168 (64)	277/566 (49)	16.3		1.83 (1.28, 2.61)	0.00080
	Total	574/906 (63)	2478/4566 (54)			1.47 (1.27, 1.71)	0.00000026
rs1495966	Dis.	467/738 (63)	2204/4000 (55)	83.4	0	1.40 (1.19, 1.64)	0.000038
	Rep.	106/168 (63)	264/548 (48)	16.6		1.84 (1.29, 2.62)	0.00070
	Total	573/906 (63)	2464/4548 (54)			1.47 (1.27, 1.70)	0.00000029
rs1966176	Dis.	506/738 (69)	2443/4000(61)	83.6	5	1.39 (1.18, 1.64)	0.00011
	Rep.	118/168 (70)	322/562 (57)	16.4		1.76 (1.21, 2.55)	0.0026
	Total	624/906 (69)	2765/4562 (61)			1.45 (1.24, 1.69)	0.0000021
rs6665569	Dis.	465/738 (53)	2210/4000 (55)	83.0	0	1.38 (1.17, 1.62)	0.000094
	Rep.	103/168 (61)	271/550 (49)	17.0		1.63 (1.15, 2.32)	0.0063
	Total	568/906 (63)	2481/4550 (55)			1.42 (1.23, 1.65)	0.0000030
rs924080	Dis.	590/738 (80)	2940/4000 (74)	86.4	62	1.44 (1.18, 1.74)	0.00022
	Rep.	143/168 (85)	413/566 (73)	13.6		2.12 (1.33, 3.37)	0.0013
	Total	733/906 (81)	3353/4566 (73)			1.53 (1.28, 1.83)	0.0000032
rs6677188	Dis.	590/738 (80)	2937/4000 (73)	85.8	34	1.44 (1.19, 1.75)	0.00019
	Rep.	142/168 (85)	419/566 (74)	14.2		1.92 (1.21, 3.03)	0.0049
	Total	732/906 (81)	3356/4566 (73)			1.51 (1.26, 1.80)	0.0000060

*SNP* single-nucleotide polymorphism, *BD* Behçet’s disease, *CI* confidence interval, *Dis.* discovery phase, *OR* odds ratio, *Rep.* replication phase

## Discussion

This study investigated the previously BD-associated *IL23R*–*IL12RB2, IL10, STAT4*, and *ERAP1* loci for fine-mapping by using comprehensive imputation for discovering candidate SNPs and genotyping them in additional cases and controls for independent replication of the association in Koreans. Among the four loci, only *IL23R*–*IL12RB2* was confirmed for association with BD susceptibility in the pooled meta-analysis of the discovery and replication phases, consistent with several previous studies [2–4, 7].

More importantly, association between BD risk and the *IL23R*–*IL12RB2* locus was fine-mapped on the intergenic region rather than the *IL23R* or *IL12RB2* gene, as the most significant association (*P* = 10^−7^) was observed with three almost perfectly correlated SNPs (*r* ^2^ = 0.99), rs1495965, rs1495966, and rs4655535 located in the intergenic region, which may contain regulatory sequences for expression of *IL23R*, *IL12RB2*, or both. These three SNPs were in complete LD (D’ = 1.0) with another intergenic SNP, rs924080, which has been associated with *IL23R* expression. More specifically, the risk-associated allele *A* of rs924080 was associated with enhanced expression of *IL23R*, *IL6*, and *TNF*α in the previous Turkish study [18], although not in the previous Chinese study [6], where *IL23R* and *IL17* mRNA levels were affected by rs12141431 instead. It is plausible to hypothesize that genetic polymorphisms in the *IL23R*–*IL12RB2* locus are associated with upregulated Th17 axis in BD. The importance of Th17 cells and cytokines in BD has been shown in many studies. Elevated levels of circulating Th17 cells or Th17 cytokines in BD have been reported [13, 14, 19, 20] and successful anti-TNFα treatment was found to decrease Th17 differentiation [15]. However, the mechanism by which the BD-associated SNPs alter disease susceptibility remains to be clarified.

Several *IL10* SNPs were positive for association with BD in the discovery phase but all failed in the replication phase. Association between BD and *IL10* was previously evident with rs1518111 in the Turkish GWAS [3] but was evident with rs1800871 and rs1800872 in the Japanese GWAS [2], whereas nominal association was observed with rs1518111 and rs1554286 in an Iranian population [7], which was not replicated in another Iranian population [8], indicating contrasting results in different studies. In this study in Koreans, the association test results were drastically different between the discovery and replication phases. This discrepancy could have been caused by heterogeneity of the enrolled subjects, among other causes. For example, the discovery phase population had significantly more eye and central nervous system lesions and fewer skin lesions than the replication phase population. It might be that the *IL10* polymorphism is preferentially involved in eye or central nervous system lesions, since the prevalence of the *IL10* polymorphism in patients with BD was higher in the discovery phase (76%) than in the replication phase (71%) whereas its prevalence was similar in controls in both phases (68% in the discovery phase vs. 71–72% in the replication phase). Another possible cause could be that the sample size was too small in the replication phase, i.e. the statistical power was insufficient. A more homogenous, larger sample will help further determine the significance of *IL10* polymorphisms in Koreans with BD.

We found no association with *STAT4* and *ERAP1*–*ERAP2* loci. Alteration of STAT4 signaling or antigen presentation of pathogenic peptides by SNPs [16] may not be a major disease susceptibility mechanism in Koreans. However, there is a possibility that the rare risk alleles may have been undetected by this fine mapping due to limited statistical power.

## Conclusion

Association was confirmed between susceptibility to BD and the *IL23R–IL12RB2* locus, and was fine-mapped on the intergenic region rather than the two flanking genes, suggesting association with altered expression of *IL23R*, *IL12RB2*, or both. The other three loci, *IL10*, *STAT4*, and *ERAP1*–*ERAP2*, were not confirmed in this study with Koreans.

## Additional file

Additional file 1
**Figure S1**. LD structure similarities of the four loci (*IL10, IL23R*–*IL12RB2*, *STAT4,* or *ERAP1*) between Korean and Asian (Chinese and Japanese) populations. Using the Korean and Asian genotype databases, the correlation coefficient value (*r* ^2^) was calculated for all the SNPs within each of the four loci in reference to the SNP found to be most significant in the Korean GWAS (rs1554286 in *IL10*, rs6677188 in *IL23R–IL12RB2*, rs1031508 in *STAT4*, and rs26652 in *ERAP1-ERAP2*). The Y axis shows *r* ^2^ between the reference SNP and the other SNPs within each gene. The X axis shows SNPs ranked in the descending order of *r* ^2^ values. In each plot, open circles represent SNPs from the Korean genotype database and solid squares those from the Asian database. (TIF 65 kb)

## Abbreviations

BD: Behçet’s disease
CI: Confidence interval
GWAS: Genome-wide association study
HWE: Hardy-Weinberg equilibrium
LD: Linkage equilibrium
OR: Odds ratio
SNP: Single-nucleotide polymorphism
Th: T helper
TNF: Tumor necrosis factor
