# Supplementary figures and images for: Behçet’s disease risk association fine-mapped on the IL23R–IL12RB2 intergenic region in Koreans
Source: Arthritis Res Ther. 2017 Oct 10;19:227. doi: 10.1186/s13075-017-1435-5 (PMC5633897; doi:10.1186/s13075-017-1435-5)

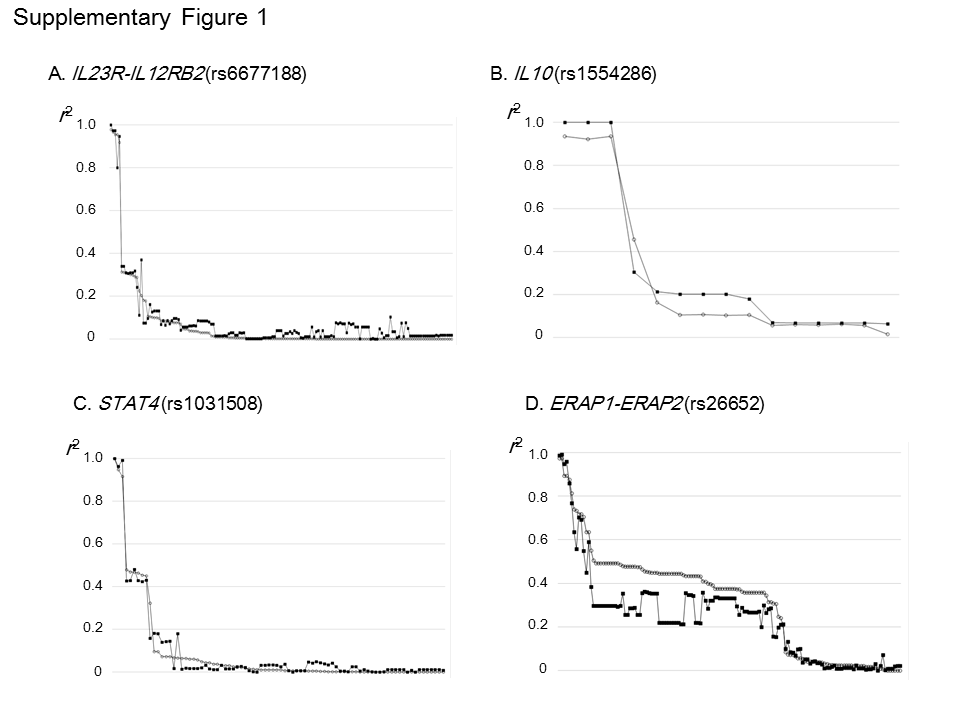

Supplement: Additional file 1 — Figure S1. LD structure similarities of the four loci (IL10, IL23R–IL12RB2, STAT4, or ERAP1) between Korean and Asian (Chinese and Japanese) populations. Using the Korean and Asian genotype databases, the correlation coefficient value (r 2) was calculated for all the SNPs within each of the four loci in reference to the SNP found to be most significant in the Korean GWAS (rs1554286 in IL10, rs6677188 in IL23R–IL12RB2, rs1031508 in STAT4, and rs26652 in ERAP1-ERAP2). The Y axis shows r 2 between the reference SNP and the other SNPs within each gene. The X axis shows SNPs ranked in the descending order of r 2 values. In each plot, open circles represent SNPs from the Korean genotype database and solid squares those from the Asian database. (TIF 65 kb) [file 13075_2017_1435_MOESM1_ESM.tif]
